# Burkholderia contaminans Bacteriophage CSP3 Requires O-Antigen Polysaccharides for Infection

**DOI:** 10.1128/spectrum.05332-22

**Published:** 2023-05-18

**Authors:** Cassandra R. Stanton, Steven Batinovic, Steve Petrovski

**Affiliations:** a Department of Microbiology, Anatomy, Physiology & Pharmacology, La Trobe University, Bundoora, Australia; b Division of Materials Science and Chemical Engineering, Yokohama National University, Yokohama, Kanagawa, Japan; CCG-UNAM

**Keywords:** bacteriophage, phage, *Burkholderia cepacia* complex, phage therapy, characterization, O-antigen, receptor, phage characterization

## Abstract

The Burkholderia cepacia complex is a group of opportunistic pathogens that cause both severe acute and chronic respiratory infections. Due to their large genomes containing multiple intrinsic and acquired antimicrobial resistance mechanisms, treatment is often difficult and prolonged. One alternative to traditional antibiotics for treatment of bacterial infections is bacteriophages. Therefore, the characterization of bacteriophages infective for the Burkholderia cepacia complex is critical to determine their suitability for any future use. Here, we describe the isolation and characterization of novel phage, CSP3, infective against a clinical isolate of Burkholderia contaminans. CSP3 is a new member of the *Lessievirus* genus that targets various Burkholderia cepacia complex organisms. Single nucleotide polymorphism (SNP) analysis of CSP3-resistant B. contaminans showed that mutations to the O-antigen ligase gene, *waaL*, consequently inhibited CSP3 infection. This mutant phenotype is predicted to result in the loss of cell surface O-antigen, contrary to a related phage that requires the inner core of the lipopolysaccharide for infection. Additionally, liquid infection assays showed that CSP3 provides suppression of B. contaminans growth for up to 14 h. Despite the inclusion of genes that are typical of the phage lysogenic life cycle, we saw no evidence of CSP3’s ability to lysogenize. Continuation of phage isolation and characterization is crucial in developing large and diverse phage banks for global usage in cases of antibiotic-resistant bacterial infections.

**IMPORTANCE** Amid the global antibiotic resistance crisis, novel antimicrobials are needed to treat problematic bacterial infections, including those from the Burkholderia cepacia complex. One such alternative is the use of bacteriophages; however, a lot is still unknown about their biology. Bacteriophage characterization studies are of high importance for building phage banks, as future work in developing treatments such as phage cocktails should require well-characterized phages. Here, we report the isolation and characterization of a novel Burkholderia contaminans phage that requires the O-antigen for infection, a distinct phenotype seen among other related phages. Our findings presented in this article expand on the ever-evolving phage biology field, uncovering unique phage-host relationships and mechanisms of infection.

## INTRODUCTION

The Burkholderia cepacia complex (Bcc) is a group of at least 24 species that are ubiquitous in nature and are highly problematic opportunistic pathogens (1–3). Consistent with the large range of ecological niches the Bcc occupies, species of the complex are also present in clinical settings and can contaminate medical equipment, including nebulizers, saline solutions, and disinfectants (4–8). The Bcc species cause chronic pulmonary infections, pneumonia, and septicemia in immunocompromised individuals, particularly those with cystic fibrosis (CF) (9, 10). Although these infections are of a smaller proportion than the pulmonary infections caused by other pathogens such as Pseudomonas aeruginosa, Bcc infections are often associated with more severe disease states and higher mortality rates (3). While chronic Bcc infections are common, Bcc patients can also rapidly develop “cepacia syndrome,” characterized by respiratory failure, necrosis, and sepsis (3). Cepacia syndrome often manifests very suddenly, and once confirmed, death is rapid (11).

Treatment of Bcc infections varies greatly depending on the strain and often requires a multidrug approach. The efficacy of antibiotics can be very limited; at best, an antibiotic is 38% effective against Bcc strains (12). The Bcc species possess large, multichromosomal genomes, ranging from 6 to 9 Mbp in total (13–15), that encode a wide range of metabolic pathways and antimicrobial resistance genes (2, 16). Intrinsically, Bcc species can express efflux pumps, catabolic enzymes, and porins for removal and protection from a large range of antimicrobials (17). As a result, Bcc infections are often problematic and prolonged due to antibiotic resistance against a wide array of antibiotics and antimicrobial peptides, including β-lactams, quinolones, tetracyclines, and trimethoprim (3, 18). The severity of Bcc infections, coupled with the difficulty to treat, has renewed interest in alternative therapies.

While the use of bacteriophages (phages) for treatment against bacterial infections is not a new concept, this past decade has seen a resurgence of interest in this field, particularly with the rise of the antibiotic resistance crisis. Phages are natural predators of bacteria, able to infect and rapidly replicate, resulting in host cell lysis. This natural process of phages can be exploited and repurposed as antibacterial therapeutics (19). Phages generally have narrow host ranges to a particular bacterial species or strain, meaning their targeted treatment will have no adverse effects on commensal microbes (20). *In vitro* work on developing both polyphage cocktails and phage-antibiotic cocktails as treatments has been promising (3, 21). However, since phages are one of the largest groups of biological entities, with an estimated 10^31^ phage particles in the biosphere (22), there is still a lot unknown about each individual phage and how they behave with their hosts.

Here, we describe novel phage CSP3, isolated against two clinical isolates of Bcc organisms, one confirmed as Burkholderia contaminans (23). CSP3 is a new member of the *Lessievirus* genus, the first to be isolated from Australia. A defining characteristic of CSP3 is that it requires the presence of the O-antigen for infection, utilizing a different receptor to other *Lessievirus* and related phages. CSP3 was able to infect and suppress the growth of a B. contaminans clinical isolate *in vitro.* Despite the presence of genes encoding the lysogenic pathway, CSP3 could be a suitable candidate for further use in treatments such as a phage cocktail.

## RESULTS AND DISCUSSION

### Isolation and morphological features of CSP3.

**FIG 1:**
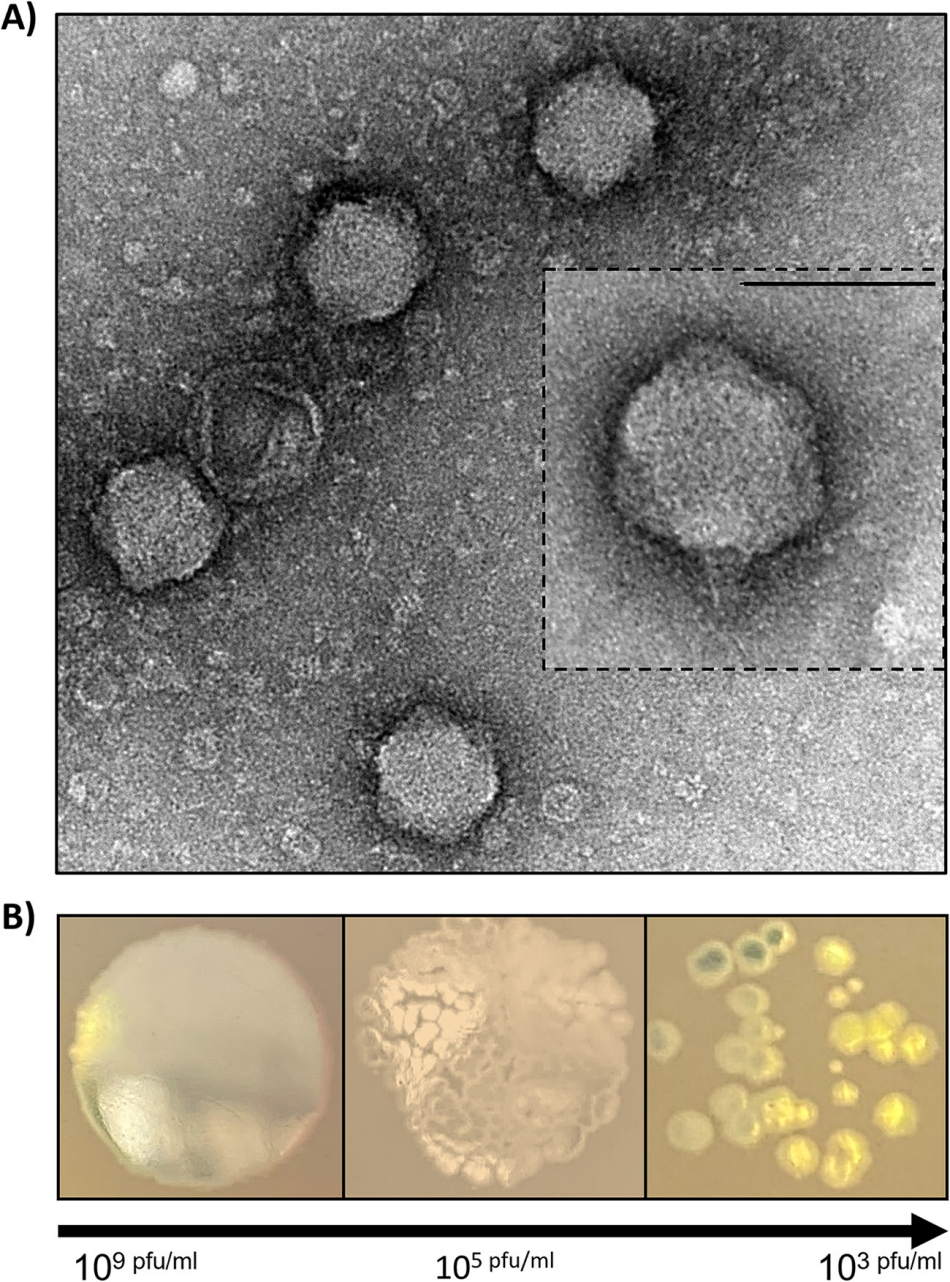
Morphological features of phage CSP3. (A) Transmission electron microscope images of CSP3 virions stained with 2% uranyl acetate. CSP3 has a noncontractile tail (15.5 ± 0.79 nm) and an icosahedral head (62 ± 0.95 nm). Tail fibers could not be visualized. Scale bar, 100 nm in the overlaid image. (B) Spot plaque assay of CSP3 filtrate on a double agar overlay with host, B. contaminans. Decreasing concentrations of CSP3 show small, individual plaques.

After screening various environmental samples, single plaques were produced on clinical isolate Burkholderia contaminans (strain 5080) (23) and another Bcc (strain 3726) from soil samples collected from Darwin, Australia. After purification and whole-genome sequencing, it was revealed that there was one distinct phage, CSP3. Transmission electron microscopy revealed CSP3 possesses a short, noncontractile tail (15.5 ± 0.79 nm) and an icosahedral head (62 ± 0.95 nm) that is typical of podovirus morphology (Fig. 1A). CSP3 produced small, clear plaques (Fig. 1B) and displayed a narrow host range, shown to only lyse the two *Burkholderia* strains and no other Gram-negative species from our culture collection. Other Bcc strains were unavailable to determine CSP3’s full host range.

### Genomic features of CSP3.

**FIG 2:**
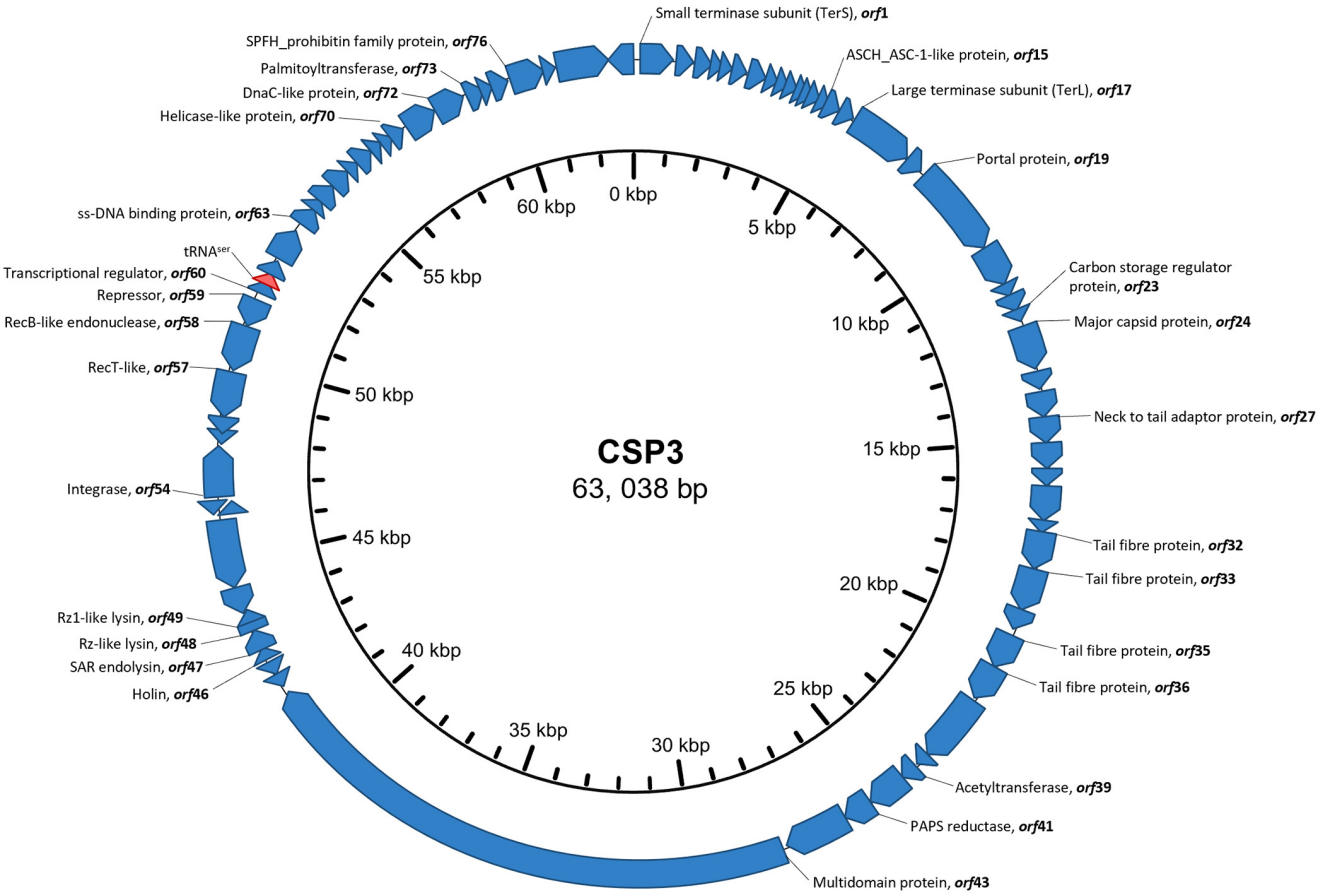
Circular genome map of CSP3. The tRNA^ser^ is colored in red, and predicted ORFs are colored in blue and numbered, with the name of the protein they encode.

The assembled CSP3 genome was 63,038 bp in length with a G+C content of 66.5% (Fig. 2). The CSP3 genome is modularly organized, with replication, lysis, structural, and lysogenic gene modules. The genome contains 79 putative open reading frames (ORFs) and one tRNA^ser^. Forty-nine of the predicted ORFs encode hypothetical proteins with unknown functions based on BLASTp analysis (Fig. 2; see Table S2 in the supplemental material). The remaining ORFs encode products involved in phage DNA replication and packaging, structural proteins, phage lysis, and prophage-related genes that suggest a lysogenic lifestyle. The CSP3 genome did not contain any obvious termini or packaging mechanisms; however, it is predicted to contain a circular permuted genome. Since there was no predicted start for the genome, we chose the small terminase gene to orientate and define the start of the genome.

**(i) Replication, packaging, and structural genes.** At the designated start of the genome is the small terminase subunit gene (*terS*), *orf1*, along with *orf17*, the large terminase subunit gene (*terL*), and the portal protein *orf19*. The insertion of 15 ORFs in between *terS* and *terL* is rather unusual, as they typically clustered closely together. However, the reasoning behind these insertions is not well known, and an insertion of this size has not been noted in other phages. Together, the TerS, TerL, and portal protein form a complex DNA packaging motor (24). TerS is predicted to recognize and bind to specific DNA packaging (*pac*) sites in the genome and regulate TerL. TerL is an ATPase that moves the double-stranded DNA (dsDNA) through the portal protein component and into the procapsid (25). Portal proteins generally form a dodecamer ring at the base of the procapsid and function in sensing when the capsid is full and controlling the shape of the capsid and maturation (26, 27). It can be hypothesized that CSP3 utilizes a headful packaging mechanism, where the terminase packages CSP3 DNA into the capsid initiated at a *pac* site that is unknown at this time.

Downstream of the packaging proteins are five structural proteins identified based on BLASTp analysis of their predicted amino acid sequence, a major capsid protein (*orf24*) that shares identity with many other described capsid proteins that contain an N4-gp56 family protein, and four genes with BLASTp matches to tail fiber genes (*o*r*f32*, -*33*, -*35*, and -*36*). Between the capsid gene and the first tail fiber gene are six hypothetical genes and *orf27*, which is a putative head-to-tail adaptor protein. *orf27* encodes an Ad3 protein superfamily domain when analyzed on the Virfam database. While there are no known functions described for the rest of these gene products, it can be assumed that some of the proteins are involved in phage structure and assembly.

The CSP3 genome contains a region of genes associated with DNA replication that begins with a putative transcriptional regulator, *orf60*. Following the transcriptional regulator and among various hypothetical genes are three genes associated with DNA replication. The first, *orf63*, encodes a single-stranded DNA (ssDNA)-binding family protein (COG0629) that is responsible for protecting DNA from nucleolytic attack, stopping the formation of secondary structures, and it also mediates interactions with downstream enzymes (28). *orf*70 encodes a putative helicase, sharing homology with the GIY-YIG nuclease family (cd00719) proteins. Helicases unwind the dsDNA for consequent replication (29). The hypothetical gene *orf71* is predicted to be a replication initiator protein by Davis et al. (30) when characterizing the phage JC1. Following this is *orf*72, which encodes a DnaC family protein (COG1484). DnaC proteins are known as helicase loaders that assist with transferring the helicase protein (31).

**(ii) Multidomain gene.** Following the structural genes and constituting approximately 22.3% of the CSP3 genome is a 13,994-bp DarB-like multidomain gene (*orf43*). This gene encodes several active domains, including a soluble lytic murein transglycosylase (MltE family, COG0741), a HepA family DNA or RNA helicase (COG0553), and an adenine-specific DNA methylase (YtxK family, COG0827). The DarB-like proteins are injected into the host cell at the point of infection and exhibit antirestriction activity to protect incoming phage DNA (32). In phage P1, they have been seen to have specific protection against EcoB and EcoK restriction systems (33). However, *darB* is functionally dependent on other proteins, including *darA*. There have been no *darA* homologues detected in CSP3 or its relatives, but it is likely they do exist. Due to the sheer size of *orf43* and its product, the conservation of this protein must provide some benefit to the phage.

**(iii) Lysis module.** The CSP3 genome contains a lysis cassette with four genes, *orf46* to *orf49*. This module follows a typical SRRzRz1 organization of phages with Gram-negative hosts that include holin (S), SAR endolysin (R), and the Rz/Rz1 spanin complex (34, 35). While the predicted product of *orf46* contains no conserved protein domains, the gene upstream encodes an endolysin and is conserved across *Burkholderia* species phages DC1, BcepIL02, BcepMigl, and Bcep22 and distantly associated (37% nucleotide identity) with a holin protein from a *Burkholderia* myovirus, Mica. Holins are required for depolarizing the cytoplasmic (inner) membrane and creating lesions or “holes” to allow for endolysins to cross the inner membrane and act on the peptidoglycan (36). No antiholin could be detected in the CSP3 genome. Following the holin is a predicted SAR endolysin encoded by *orf47*, with a conserved lyz_P1 domain (cd16901). Endolysins are secreted into the periplasm, triggered by holins, where they degrade (36). Finally, the lysis cassette ends with the genes encoding Rz/Rz1-encoding proteins *orf48* and *orf49*, with *orf49* embedded within *orf48*. The Rz gene contains a conserved Phage_lysis (pfam03245) domain. Rz/Rz1 forms a complex that disrupts the outer membrane of the host bacterium (34, 35).

**(iv) Lysogenic lifestyle-associated genes.** Downstream of the lysis module and separated by two hypothetical proteins are four genes associated with prophage formation and the phage lysogenic life cycle, *orf54* and *orf57* to *orf59*. The integrase, *orf54*, is transcribed in the reverse orientation of the other genes associated with lysogeny and shared homology to other phage proteins containing an INT_Rci_Hp1_C (cd00796) conserved protein domain. It is predicted to be a tyrosine recombinase that facilitates site-specific recombination of the phage genome into the hosts (37). A putative repressor, encoded by *orf59*, is the first transcribed gene out of the five genes (two of which are hypothetical) on the negative strand and is predicted to function similarly to the lambda cI repressor, allowing lysogenic conversion (38). The other three genes described to aid in phage recombination events are a RecT-like protein, *orf57*; a RecB-like endonuclease, *orf58*; and a repressor, *orf59*. The RecT works in conjunction with an endonuclease to bind to single-stranded DNA overhangs that have been generated by the endonuclease and assist in the single-stranded pairing (39). Adjacent to the repressor encoding gene on the positive DNA strand is a gene encoding a putative transcriptional regulator protein, Orf60, assumed to repress lysogenic conversion and a tRNA^ser^ (38).

**(v) Moron genes.** CSP3 contains six described ORFs that encode proteins not of phage origin, termed “moron genes.” Inserted in between the terminase subunit genes is *orf15*, encoding a gene with homology to bacterial ASCH domain-containing proteins. ASCH domain proteins can aid in RNA binding and coactivation, RNA processing, and translation regulation (40). Upstream of the predicted major capsid protein is *orf23*, encoding a protein that shares homology with carbon storage regulator family domain (CsrA) proteins. CsrA proteins are associated with a wide range of functions, including carbon storage, secondary metabolism, and virulence of plant and animal pathogens due to their RNA-binding properties (41, 42). Downstream of the tail fiber genes is a gene encoding an *N*-acetyltransferase (*orf39*) with a RimL family domain (COG1670) and *orf41*, a 3′-phosphoadenosine 5′-phosphosulfate sulfotransferase (PAPS reductase) with a CysH family domain (COG0175). Phage-encoded acetyltransferases have been shown to reduce the activity of bacterial RNA polymerase, leading to a decline in bacterial transcription and freeing up resources for phage genome replication and transcription (43). PAPS reductases function to assimilate inorganic sulfate for bacterial metabolism. While this could be a selective advantage for the host bacteria, in Escherichia coli phage 186, the encoded PAPS reductase was found to be nonessential (44). *orf72* encodes a protein that shares amino acid similarity to palmitoyltransferase (PagP)-like proteins. PagP is responsible for increasing bacterial fitness and virulence by modifying the lipid A component of the Gram-negative cell wall (45). However, it was recently demonstrated in Salmonella that several virulence genes, including *pagP*, were downregulated upon exposure to phage (46). The final moron gene and exclusive to CSP3, *orf75*, encodes an SPFH_prohibitin domain (HlfC family; COG0330) protein. HlfC is a part of the *hfl* locus that modulates the lifestyle of lambda phage. HflC, along with HflK, forms a protein complex that modulates the activity of HflB, which directly inhibits lambda phage protein CII, which drives the lysogenic life cycle (47). Therefore, the presence of phage-encoded HlfC could help drive lysogeny in CSP3. However, no homology of the other genes in the *hfl* locus is present in the CSP3 genome, and HflC requires the presence of HflK for correct protein folding (48).

Without further investigation, it is difficult to determine if these moron genes are functional when expressed from CSP3. Moron genes are typically expressed during the lysogenic life cycle when a phage is integrated, and the majority of the genome is repressed (49). While the moron gene functions remain unknown, we cannot dismiss their potential increase in bacterial virulence, particularly *orf72* and *orf75*. However, the addition of *orf15* and *orf39* in the CSP3 genome may be more beneficial to phage replication processes.

### Whole-genome comparisons and classification of CSP3.

For classification of CSP3, a combination of whole-genome identity, gene sharing, and phylogenetic analysis was undertaken. First, complete phage genomes that matched to CSP3 on the GenBank database were collated for analysis. Any bacterial genomes considered to contain prophages and genomes that matched <15% coverage were not used. A total of 19 complete genomes, including CSP3, were collated, five described as phages infective for other Burkholderia cepacia complex organisms and the others infective against *Ralstonia* spp.

**FIG 3:**
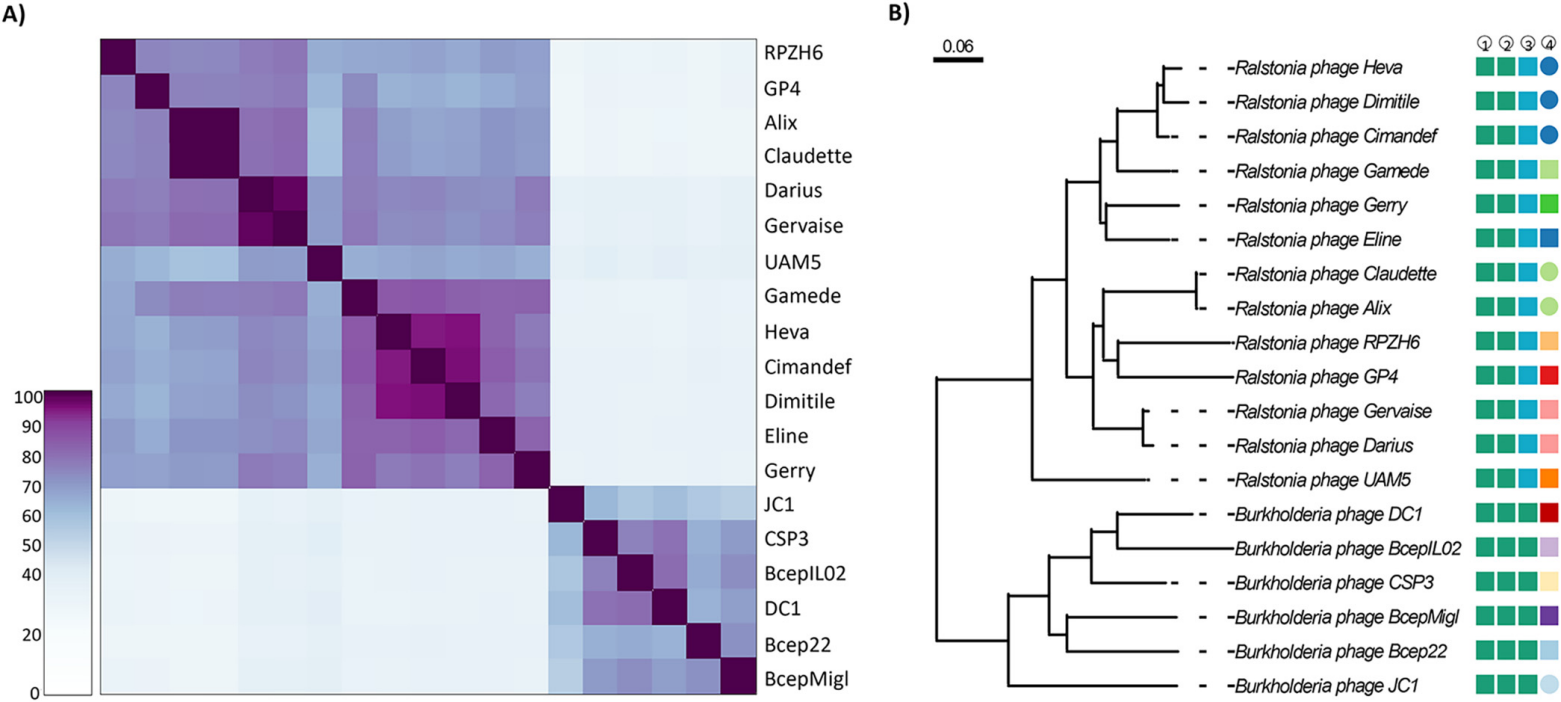
Whole-genome and phylogenetic relationships between CSP3 and related phages. (A) Intergenomic similarities between CSP3 and the top phage matches on the NCBI database. The blue and orange dashed-line boxes indicate the two separate phage groups. (B) Whole-genome (amino acid) phylogenetic tree using GBDP *d*_6_ formula with suggested taxa collated with data from the ICTV. (1) Family-level classification; (2) subfamily-level classification; (3) genus-level classification; (4) species-level classification.

**(i) Whole-genome and phylogenetic investigations.** Intergenomic identities were generated to compare the relatedness between CSP3 and the *Burkholderia* and *Ralstonia* phages. CSP3 clusters tightly with *Burkholderia*-infecting phages Bcep22, BcepIL02, BcepMigl, DC1, and JC1 (Fig. 3A). CSP3, along with the *Burkholderia* phages, shares a more distant relationship to the *Ralstonia* phages, sharing approximately 35% intergenomic identity. Within the *Burkholderia* phage cluster, there is divergence between JC1, which shares approximately 60% intergenomic identity with the other phages, and CSP3. CSP3 represents a novel species of this phage cluster, as it is genetically distinct, sharing <95% nucleotide similarity to the other phages. CSP3, DC1, and BcepIL02 cluster together within the *Burkholderia* phage group, sharing more nucleotide identity than the other phages. A proteome-scale phylogenomic tree using VICTOR (50) was generated to explore the groupings further (Fig. 3B). As anticipated, there was a divergence between the *Burkholderia* phages and the *Ralstonia* phages. Within the *Burkholderia* phage cluster, CSP3, DC1, and BcepIL02 branched from BcepMigl and Bcep22 in their own separate clades, whereas JC1 formed its own clade.

**FIG 4:**
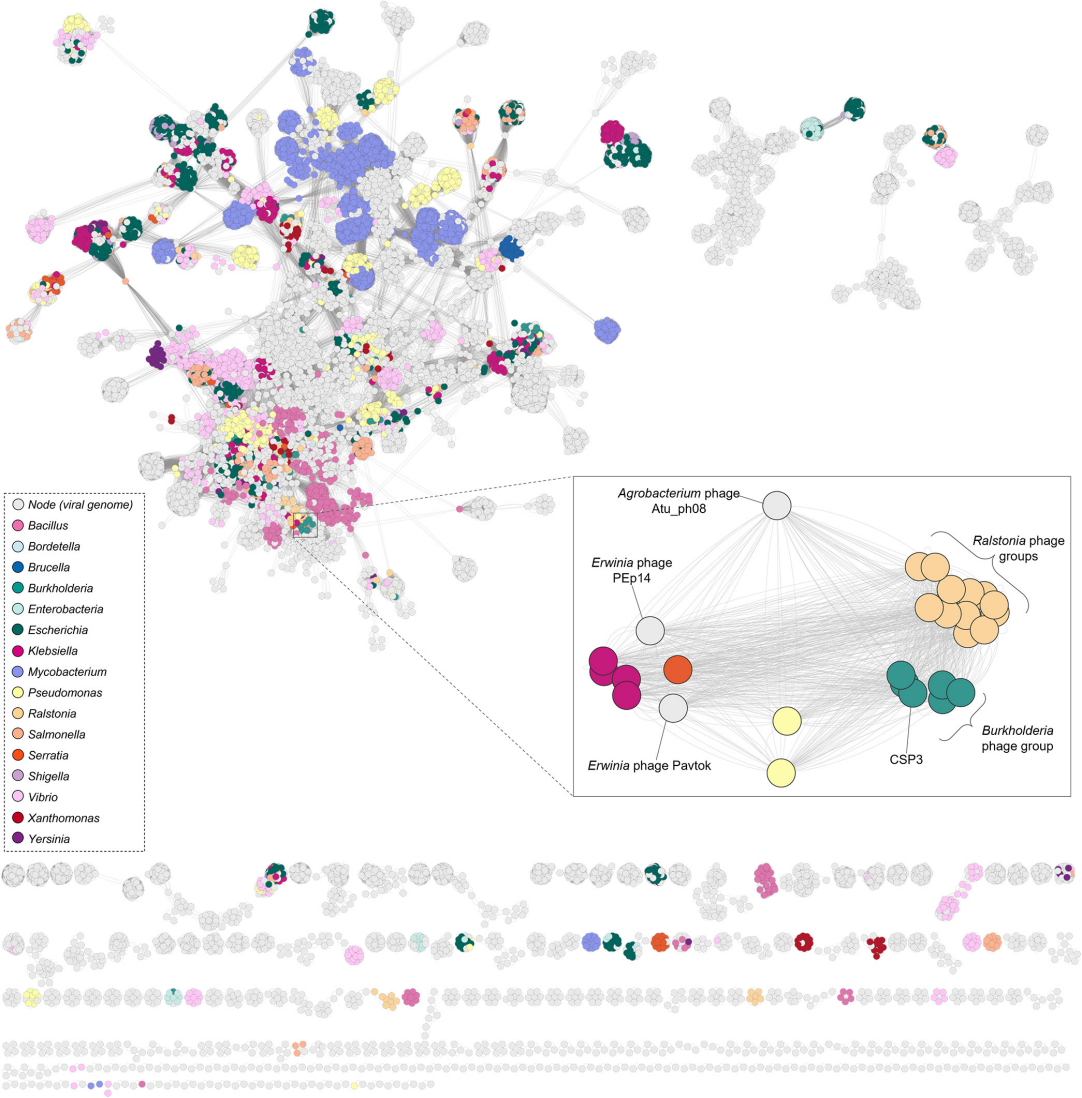
Gene sharing and placement of CSP3 and relatives. A reticulate network of CSP3 and over 17,000 phage genomes (nodes) currently deposited on the GenBank database with key bacterial host genera color coded to highlight their placement. The zoomed in panel shows CSP3 with the *Burkholderia* phage group, *Ralstonia* phage group, and other relatives using an edge-weight spring-embedded layout model to highlight their gene sharing, indicated by the lines (edges) connecting the nodes.

To uncover the gene sharing among the *Burkholderia* phage group and the *Ralstonia* phages and positioning among other phages, a vConTACT2 (51) reticulate network was generated. Figure 4 depicts the global network of all phages currently deposited to GenBank as of August 2022 and their positioning with CSP3. There are currently 17,694 individual phages (termed nodes), including CSP3. Highlighted by colors are some of the key bacterial genera, in particular, Gram-negative bacteria related to *Burkholderia*. CSP3 and the *Burkholderia* phage group are clustered tightly together and positioned among the *Ralstonia* phages as predicted, but also with other Podovirus phages infective for Klebsiella, Pseudomonas, and *Erwinia*. There are 812 individual gene-sharing events within the CSP3-specific network. One example of gene sharing was the dispersion of the DarB-like antirestriction protein, found to be highly conserved within the *Burkholderia* and *Ralstonia* phage groups. The ICTV has currently classified phages Bcep22, BcepIL02, BcepMigl, and DC1 into the *Lessievirus* genus. Since CSP3 contains a distinct genome <95% similar to another phage, CSP3 can be classified as a novel species within the *Lessievirus* genus (52). While JC1 has conservation and similarities to these *Lessievirus* phages, it is <70% similar to the other *Burkholderia* phages and could represent a novel genus.

**FIG 5:**
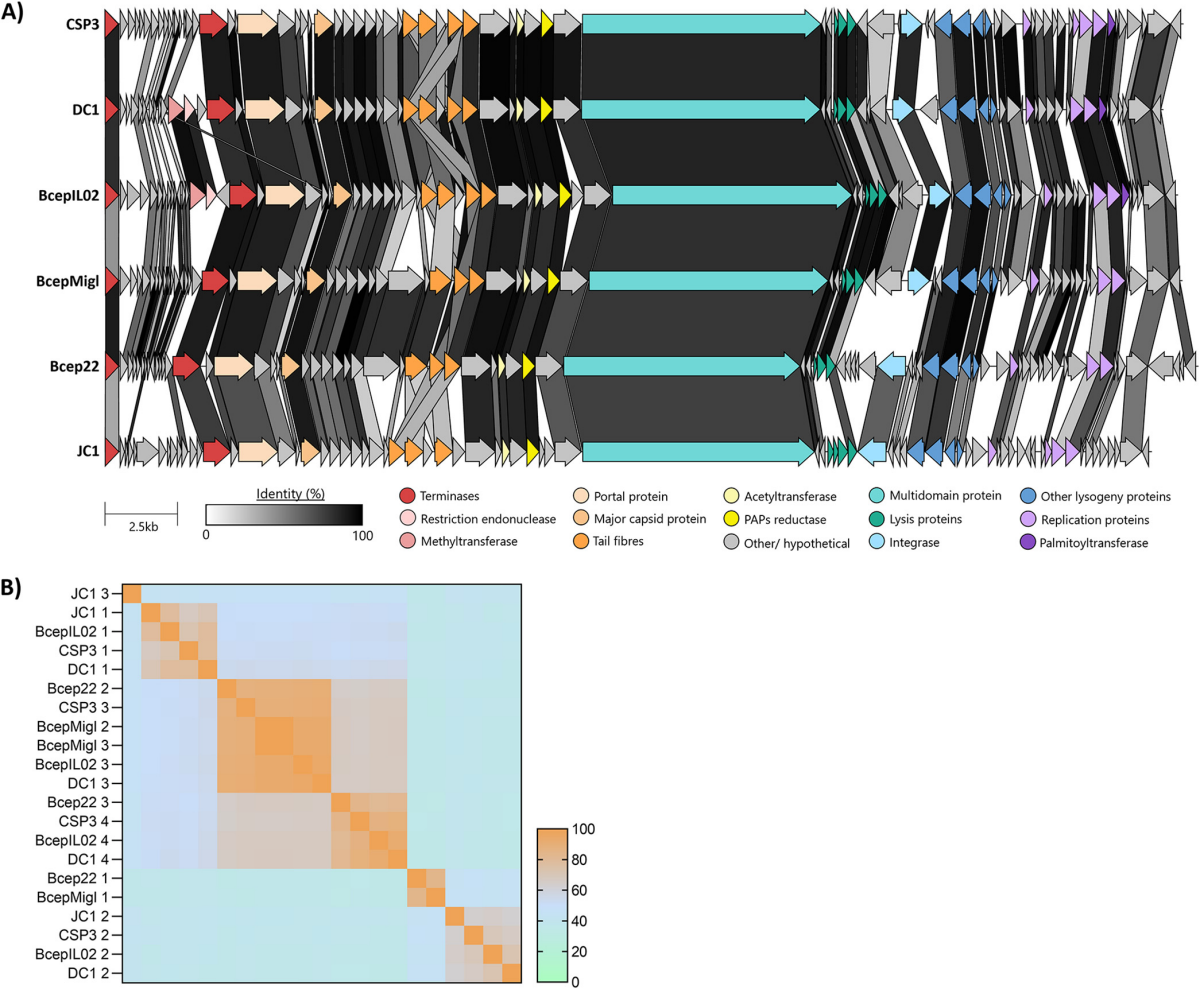
Whole-genome comparison of the *Lessievirus* group and CSP3. (A) Whole-genome map alignments of the *Lessievirus* genus and CSP3, aligned from the small terminase gene. Key genes are color coded, and identity of shared percentage is shown by the grayscale bar. (B) Heatmap comparison of the shared amino acid identities of the *Lessievirus* group tail fiber genes (TFGs). Each TFG is numbered 1 to 3 or 4 in order of how they appear within the genome (i.e., CSP3 TFG 1 is labeled as CSP3 1).

**(ii) Genome structure and conservation between CSP3 and other *Burkholderia* phages.** To investigate the genome conservation of CSP3 with the other *Burkholderia* phages, whole-genome map alignments were generated. These six phage genomes all contained a very conserved genome structure (Fig. 5A). However, there are a few notable regions of dissimilarity between CSP3 and the five other phages, including the genes between the small and large terminase genes, the tail fiber genes (TFGs), and in the modules with lysogeny and DNA replication. CSP3 contains small insertions of <5 kb throughout its genome, notably in between the small and large terminase subunits, that differ from the other phages. There are 15 predicted coding DNA sequences (CDSs) between the two terminase subunits, half of which are shared between CSP3 and its relatives, while the other half are unique to CSP3 and share homology to bacterial genomes, mainly *Burkholderia* spp. All gene products except one, Orf15, contain no predicted function or conserved domains and are noted as hypothetical. Orf15 contains an ASCH_ASC-1_like conserved domain and is unique to CSP3. Within this region, the two closest relatives to CSP3, DC1 and BcepIL02, both contain a DNA methyltransferase gene (COG0863) and a restriction endonuclease, whereas CSP3 does not.

A unique feature of this group of phages is that their genomes contain multiple copies of tail fiber genes (TFGs). CSP3, DC1, and BcepIL02 contain four annotated TFGs; however, phages BcepMigl, Bcep22, and JC1 only have three. To investigate the homology of the multiple tail fiber genes, a Clustal Omega pairwise comparison of each individual phage TFG (in total, 21 genes) was produced (Fig. 5B). The heatmap shows that, generally, each numbered TFG clusters together in amino acid similarity (i.e., all the TFGs that appear first in the genome, noted as TFG1, cluster together). However, the phages that contain only three TFGs cluster in a different pattern. The TFG1s of Bcep22 and BceMigl cluster together, sharing 84% similarity, and are approximately 45% similar to the TFG2s of JC1, CSP3, BcepIL02, and DC1. BcepMigl TFGs 2 and 3 and Bcep22 TFG2 are more similar to CSP3, BcepIL02, and DC1 TFG3, whereas Bcep22 TFG4 is more related to CSP3, BcepIL02, and DC1 TFG3. We hypothesize that Bcep22 and BcepMigl lost their original TFG1 and that their described TFG1 is actually a more modified version of the other phages’ TFG2. JC1 is particularly unique, as its TFG3 is very distinct from the other phages’ tail proteins and its own. The addition of this protein may explain JC1’s ability to also infect a different genus, *Ralstonia*, and its use of a different host receptor from CSP3. It is evident that the TFG region is prone to genetic recombination and organization, over time leading to multiple genetically distinct genes that share a core homology. While there is not a lot known about phages maintaining multiple tail fiber genes, it appears that it may be an advantage to increasing host range. While all the TFGs have the potential to be expressed, it would be highly unlikely that all three or four TFG products are assembled onto each virion. As discussed by Gill et al. (38), it is more likely that not all the genes are functional and redundant and that they are all expressed but assembled into different virions, creating a heterogenous population of virions. This could explain the group's broad host range and variations in efficiency of plating, depending on species.

CSP3 and JC1 represent the only two members of this *Burkholderia* phage group to contain distinct replication module structures. The inclusion of a helicase and the “helicase-initiator-helicase loader” structure displayed in CSP3 and JC1 differs from an “initiator-helicase loader-helicase” structure seen within the other phages of this group. Phage genomes are typically mosaic in nature and are constantly being reshaped by horizontal gene transfer events, particularly in lysogenic phages (53). Random additional DNA can also be integrated within the genome due to the non-sequence-specific cuts made by the large terminase (54). These factors may explain the variability in the region of the genome between the small and large terminase subunits.

### Biological properties of CSP3.

**FIG 6:**
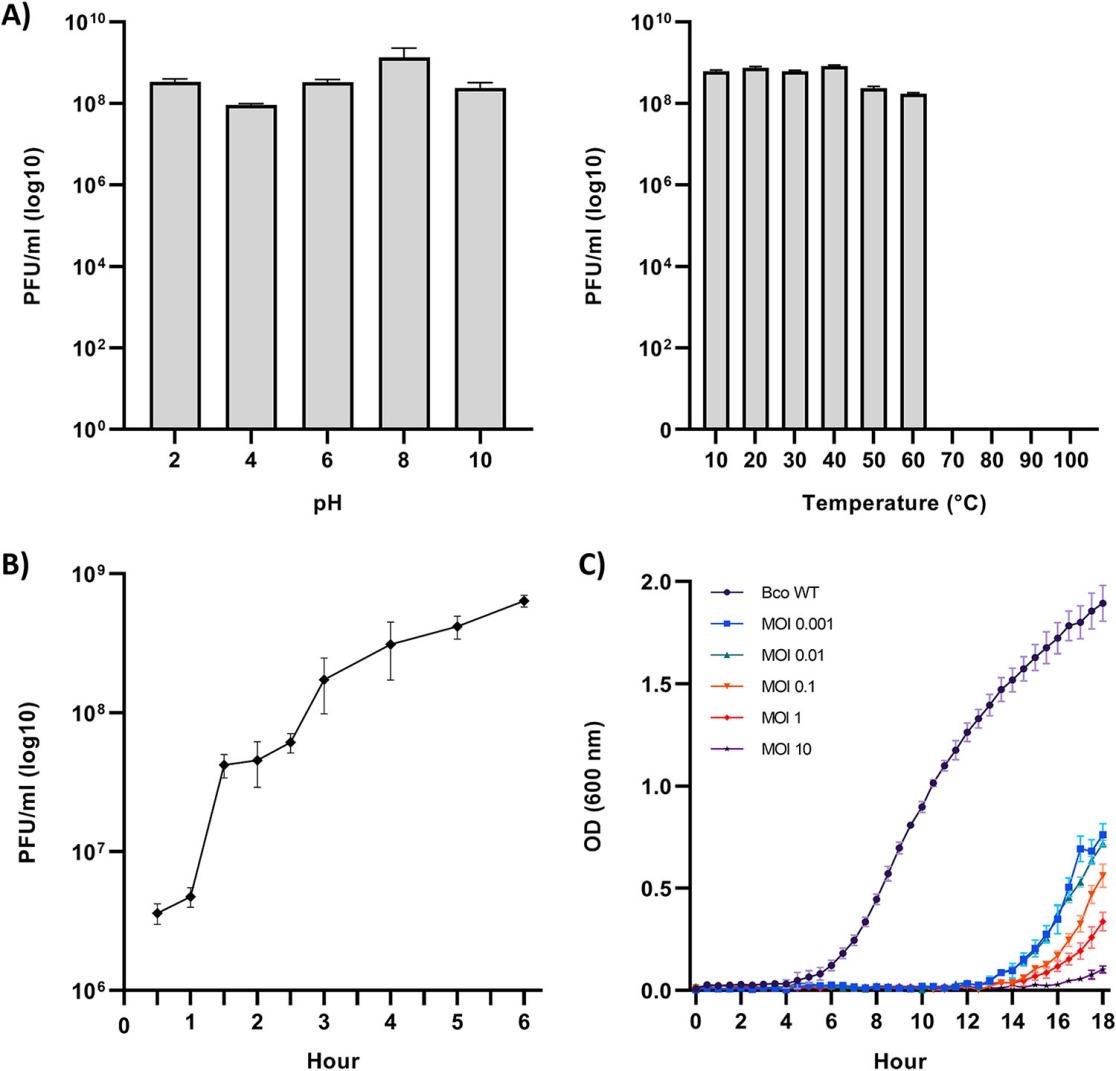
Biological properties of CSP3. (A) pH and thermal stability of CSP3. (B) Growth curve of CSP3. (C) Bacterial growth reduction assays using multiple MOI ratios of B. contaminans (Bco) and CSP3 over 18 h. Panels A to C are presented as the means of three biological replicates with error bars representing SEM.

CSP3 was tested for its stability at various pH levels and temperatures (Fig. 6A). CSP3 virions displayed insensitivity to a large pH range of 2 to 10, not significantly dropping in PFU per milliliter. It is expected to see phages found in highly acidic environments such as fermented foods and gastrointestinal tracts to be stable at low pH (55–57), not from soil environments. However, the region of the soil from which CSP3 was isolated typically ranges from pH levels of 4 to 6 (58), and it is probable that CSP3 virions are adapted to these acidic conditions. To corroborate this finding, a metagenomics study found that soils which had low pH were highly abundant in *Lessievirus* phages (59), further justifying that CSP3, and potentially *Lessievirus* members, can tolerate low-pH environments. At temperatures of 10°C to 60°C, CSP3 remains stable and viable. However, once temperature surpasses 60°C, CSP3 is no longer viable.

A growth curve determined CSP3 to have a latency period of an hour and an average burst size of 177 phages at 6 h (Fig. 6B). A range of multiplicity of infection (MOI) ratios of CSP3 and B. contaminans was tested to see the optimal MOI for reducing bacterial growth (Fig. 6C). B. contaminans growth was well suppressed by CSP3 at all MOIs tested up until 14 h, where bacterial growth did begin to slowly recover. At 18 h, B. contaminans growth reached an optical density at 600 nm (OD_600_) of 1.9, whereas with CSP3 present, the B. contaminans OD_600_ remained under 0.5 at all MOI ratios. While these are promising results *in vitro*, further usage of CSP3 therapy to clear B. contaminans would need to be studied further.

### CSP3 lifestyle.

Previously published *Lessievirus* phages have been shown to be not strictly lytic but also not lysogenic. While they contain genes that would suggest lysogeny, it has been found that they form “unstable lysogens” that are degraded before complete insertion into their host genome (38). However, Davis et al. (30) recently found that JC1, a relative of CSP3, can form stable lysogens. With the current diversity of lysogenic and unstable lysogenic phages relating to CSP3, the ability exists for CSP3 to integrate into B. contaminans genome. Screening of overnight cocultures of B. contaminans with CSP3 indicated that no CSP3 lysogens were present. Gill et al. (38) studied the ability of Bcep22 and BcepIL02 to form stable lysogens in their host strains. They hypothesized that although the lysogenic genes could be functional, stable prophages could not integrate into the host genome. Prophages may be formed but cannot integrate and are consequently lost during bacterial cell division. Since we found no evidence of lysogeny, we hypothesize that CSP3 behaves similarly to the other *Lessievirus* phages and produces unstable lysogens during infection of B. contaminans.

### Determination of CSP3 receptor.

**FIG 7:**
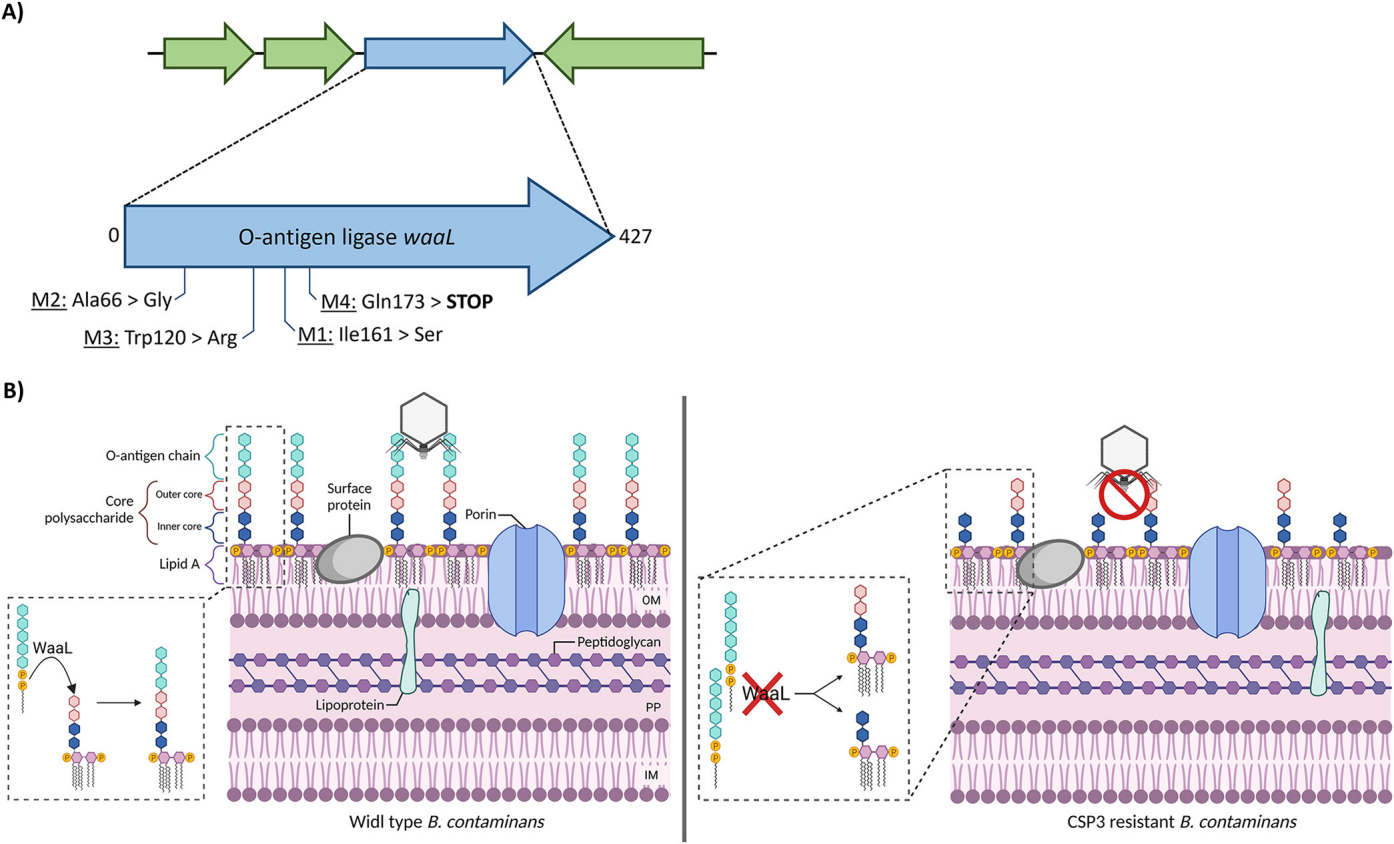
B. contaminans *waaL* gene mutations and predicted modification of the LPS. (A) *WaaL* mutations found in four CSP3-resistant mutants. (B) Schematic of the changes to the cell wall LPS upon mutation of the *waaL* gene and effects on CSP3 infection. The left panel depicts regular cell wall and LPS composition, compared to the right panel, which depicts the putative phenotype in the mutant B. contaminans. Mutation to WaaL and loss-of-function results in the O-antigen and, potentially, the outer core polysaccharide unable to attach to the lipid A and outer core polysaccharide. This results in CSP3 unable to attach and, ultimately, unable to lyse its host.

Previous work on the *Lessievirus* phages had not included resolving the host receptor required for infection until only recently. JC1, a close relative to the *Lessievirus* phages and CSP3, was found to require the inner core of *Burkholderia* species lipopolysaccharides (LPSs) for infection (30). Since JC1 is more genetically distinct, particularly within the tail fiber genes, we wanted to compare any differences between receptors of JC1 and CSP3. Five B. contaminans 5080 colonies (named M1 to M5) that gained spontaneous resistance to CSP3 infection were sequenced to determine any genotypic differences from the wild-type (WT) B. contaminans. Four of the five colonies contained distinct mutations within the O-antigen ligase gene, *waaL* (Fig. 7A). B. contaminans M1, M3, and M4 all contained a single nucleotide polymorphism (SNP) within *waaL* that resulted in a substitution of amino acid. M5 contained identical mutations to M1 and was therefore disregarded in further analysis. While M1 and M3 resulted in missense mutations that did not affect the rest of the protein translation, the substitution in M4 caused a nonsense mutation, introducing a stop codon and truncation in WaaL. B. contaminans M2 contained a single base pair insertion that caused a frameshift mutation and truncation of WaaL, resulting in the amino acid translation diverging from the WT by 71.08% and shortening of the protein by 391 amino acids.

WaaL is an integral membrane protein that mediates the attachment of the O-antigen to the lipid A core of LPS. Previous work in Pseudomonas aeruginosa, a close relative of *Burkholderia*, showed that the cell walls of *waaL* knockouts were devoid of semirough LPS (that contains no O-antigen but the outer core of the core polysaccharide) and both the A- and B-band O-antigen polysaccharides (60). Several essential residues have already been determined for WaaL function, including Arg186, Arg209, Ag268, Arg288 His319, and His337 in E. coli (61, 62), Arg186 and His311 in Vibrio cholerae (63), and His303 in P. aeruginosa (60). B. contaminans WaaL may contain similar important residues such as the ones uncovered here (Ala66, Trp120, Ile161, and Gln173) that are also essential for WaaL structure and function. Comparison of B. contaminans WaaL with the 100 top BLASTp hits revealed that all four residues where the mutations occurred remained 100% conserved. We hypothesized the genetic variations to *waaL* in this study resulted in loss of function, protein instability, or disruption of the active site of WaaL. This led to incorrect formation of the LPS with the O-antigen unable to attach to core polysaccharide and/or the production of rough LPS (that contains only the inner core polysaccharide), ultimately preventing CSP3 from infection (Fig. 7B). The requirement of CSP3 utilizing the O-antigen for infection differs from the receptor of JC1. JC1 was found to use the inner core of the LPS for infection and still was able to infect B. cenocepacia knockout strains that lacked the outer core and O-antigen (30). This difference may arise from their distinct tail fiber genes, particularly TFG 3 in JC1 that shares little homology to CSP3 TFGs.

Due to limited access to other Bcc strains to test the CSP3 host range, we compared the O-antigen gene cluster of CSP3 host B. contaminans 5080 to other Bcc strains within the GenBank database to observe the conservation of this region among Bcc strains and predict CSP3 host range. Four B. contaminans strains shared 100% nucleotide identity to the CSP3 O-antigen gene cluster, and four other B. contaminans strains shared between 99.99% and 99.97% nucleotide identity to B. contaminans 5080. When comparing multiple Bcc species, BLAST coverage ranged between 81% to 49%, and nucleotide identity ranged from 95.41% to 89.35% across eight species of Bcc. We can hypothesize that CSP3 may be able to infect the four B. contaminans strains that have 100% conserved O-antigen gene clusters; however, it is harder to predict solely based on sequence data what impact divergence to the O-antigen cluster would have on CSP3 infectivity.

The modification of B. contaminans LPS may be a disadvantage to bacterial fitness and virulence. It has been shown that inactivation of Klebsiella pneumoniae WaaL results in significant reduction in capsule retention and decreased bacterial virulence when tested in a murine model (64). However, in P. aeruginosa and B. cenocepacia, O-antigen modification is a common adaption technique to avoid host detection during chronic CF lung infections (65, 66) and also affects swimming and twitch motility (60). The virulence of O-antigen biosynthesis-deficient Yersinia enterocolitica is also affected, demonstrating downregulation of the expression in some attachment and invasion virulence genes (67). Future use of phages for antimicrobial treatments should consider phage-host interactions, the receptors required for phage infection, and what consequences of host mutation will be. While there can be both negative and positive consequences to B. contaminans LPS modification, utilizing CSP3 in a phage cocktail or with other antimicrobial agents may decrease the chance of phage resistance, provide broader-spectrum activity against other Bcc strains, and improve treatment outcomes.

### Conclusion.

This study characterized CSP3, a novel *Lessievirus* phage, the first to be isolated from Australia, that targets two Burkholderia cepacia complex clinical isolates. CSP3 provided excellent suppression of B. contaminans growth, and although its genome contains genes indicative of lysogeny, there was no evidence of integration into the B. contaminans genome to suggest lysogeny was occurring. CSP3 is the first member of the *Lessievirus* genus to demonstrate its requirement for O-antigen on the LPS in B. contaminans. This differs from the related phage, JC1, that was found to require the inner core of the LPS. CSP3 could be a valuable tool for treatment of Bcc infections; however, its full host range would need to be confirmed. Further work characterizing phages targeting clinically important bacteria such as the Bcc is critical in the need for alternative antimicrobials. This study also highlights the need for phage characterization to uncover differences in host infection mechanisms that could otherwise be overlooked.

## MATERIALS AND METHODS

### Bacterial strains and growth conditions.

Bacterial and phage strains were grown in LB broth (10 g L^−1^ tryptone, 5 g L^−1^ yeast extract, and 10 g L^−1^ sodium chloride) and LB agar (with added 12 g l:1 agar) at 37°C. Medium components were from Oxoid, sourced from Thermo Fisher Scientific, Australia.

### Isolation and purification of phages.

Soil samples collected within a 30-km radius of Darwin, Australia, were used to screen for putative phages. The soils were combined with an equal volume of Milli-Q water, vortexed, and centrifuged at 6,000 × *g* for 10 min. Liquid samples were centrifuged at 6,000 × *g* for 10 min. The supernatants were filtered through a 0.22-μm pore filter to remove any bacteria. Then, 200 μL of the filtered environmental samples was added to active-growth-phase *Burkholderia* cultures. The cultures were incubated at 37°C overnight. Following incubation, the enrichment culture was centrifuged at 12,000 × *g* for 5 min, and the supernatant was filtered through a 0.22-μm pore filter. Soft agar overlay plates of *Burkholderia* spp. were prepared with 100 μL of the enrichment filtrate and 1 mL of bacterial culture. Following overnight incubation, any clearings were presumed plaques and underwent three rounds of single plaque purification.

### Transmission electron microscopy.

Copper grids (ProSciTech, Australia) coated with carbon and Formvar were subjected to a glow discharge treatment for 60 s. Phage filtrates (>10^9^ PFU/mL) were briefly centrifuged before staining to remove small cell debris that can pass through the 0.22-μm filter. Ten microliters of the phage filtrates was allowed to absorb onto the surface of the grids for 60 s, followed by removal of excess residue with filter paper. The grids were washed twice by spotting 5 μL of Milli-Q water and then negatively stained with three 3-μL spots of 2% (wt/vol) uranyl acetate that were removed immediately. After staining, one final Milli-Q wash was performed, and the grids were dried at room temperature for 30 min. The grids were examined under a Jeol JEM-2100 electron microscope at 200 kV. Images were processed on ImageJ (Fiji) v1.53c to measure capsid and tail lengths.

### Phage DNA extraction, genome sequencing, and annotation.

Purified phage particles were precipitated using polyethylene glycol (PEG) followed by a proteinase K treatment to extract DNA as described previously (68). Isolated phage DNA (100 ng) was prepared using the NEBNext Ultra II DNA library prep kit (NEB) followed by whole-genome sequencing on an Illumina MiSeq v2 300-cycle kit with 150-bp paired-end reads. Raw data were filtered using Trim Galore v0.6.4 with the default settings (Q scores of ≥20, with automatic adapter detection) (69). Phage genomes were assembled using SPAdes v3.12.0 with default settings (70).

The phage genomes were manually screened for putative open reading frames (ORFs) using Geneious 11.1.5 and Glimmer (71) on default settings. Sequence similarity searches were conducted using the predicted amino acid sequences against the GenBank database, and the BLASTp algorithm was used (72, 73). Structural genes were analyzed on the Virfam database (74). Conserved domains and motifs were identified using the conserved domain database (CDD) (http://www.ncbi.nlm.nih.gov/Structure/cdd/cdd.shtml) and the Pfam database (http://pfam.sanger.ac.uk) (75). The presence of any tRNA molecules was screened for using ARAGORN (http://www.ansikte.se/ARAGORN/) (76). Analysis of genome termini and mode of packaging was completed using PhageTerm (Galaxy v1.0.12) using standard parameters (77). Once annotated, the genome was visualized using GView Server v1.7 (https://server.gview.ca/) (78).

### Whole-genome and phylogenetic comparisons.

The top BLASTn matches against CSP3 in the GenBank database were retrieved for analysis. For genomic similarity comparisons, sequences of 19 phages, including CSP3, were collated into a singular FASTA file that was then analyzed using VIRIDIC v1.0 (http://rhea.icbm.uni-oldenburg.de/VIRIDIC/) (79). The genome map comparisons were generated using Clinker v0.0.23 (80). For gene content and sharing analysis, vContact2 was used (51). Briefly, the CSP3 genome was collated into a single amino acid FASTA file with >17,000 phage genomes from the GenBank database as of August 2022, from inphared (81). Gene2Genome was used to assign and map the protein sequences for input into vContact2. The output network was then visualized in Cytoscape v3.9.1 using the preferred layout for the global map and an edge-weighted spring-embedded model layout for the smaller cluster of phages studied. Finally, whole Genome BLAST Distance Phylogeny (GBDP) tree was generated using VICTOR web software (50). The *d*_6_ distance values formula was used, and the tree was visualized in iTOL v6 (https://itol.embl.de/) (82).

### Liquid infection assays.

To determine CSP3’s ability to lyse B. contaminans, liquid infection assays were completed. An overnight culture of B. contaminans was adjusted down to an OD_600_ of 0.05, and then a serial dilution to 10^−9^ was plated to determine a corelating colony count. At an OD_600_ of 0.05, B. contaminans culture contains 1.2 × 10^8^ CFU/mL. For the setup of the assays, a 24-well culture plate was set up in technical duplicates of a negative control (LB), a positive control (LB plus B. contaminans), and a mixture of B. contaminans and CSP3 at an MOI of either 0.001, 0.01, 0.1, 1, or 10. The plates were incubated for 16 h at 37°C in a CLARIOstar microplate reader with OD_600_ values taken every 30 min. Results were graphed in GraphPad Prism 9, and the assays were completed in biological triplicates.

### One-step growth curve.

Using CSP3, a one-step growth curve was done as described by Davis et al. (30) with modifications. Briefly, B. contaminans cultures were grown overnight and diluted down to an OD_600_ of 0.05. CSP3 was then added to B. contaminans to an MOI of 0.1, incubated at 37°C for 10 min, and then pelleted and washed with 200 μL of LB to remove any unabsorbed phages. The washed pellet was then resuspended and added to 5 mL of LB and grown at 37°C with shaking. One hundred microliters of culture was taken every 30 min for the first 3 h and then every hour for the final 3 h (6 h total), including time point 0. Each sample was immediately centrifuged for 5 min to pellet bacteria, 10 μL of the supernatant was taken, and a serial dilution was performed to 10^−6^. Dilutions were plated out on soft agar overlays of B. contaminans, and PFU per milliliter for each time point were calculated. The resulting PFU per milliliter were analyzed on GraphPad Prism 9. Burst size was calculated using the following formula: burst size = (average titer of phages at plateau time point)/(average titer phages at end of lag time point). This was completed in three biological replicates.

### Temperature and pH sensitivity assays.

For pH sensitivity, LB broth was prepared, and pH was adjusted to 2, 4, 6, 8, and 10 using either 10 M NaOH or high-concentration HCl, sterilized, and allowed to cool to room temperature. One hundred microliters of CSP3 (1 × 10^10^ PFU/mL) added to 10 mL pH-adjusted LB was mixed and incubated at room temperature (average temperature of 23.2°C) for 1 h. Serial dilutions were then completed and plated on soft agar overlays to determine PFU per milliliter.

To assess CSP3’s ability to survive different temperatures, 100 μL of CSP3 filtrate (~1 × 10^8^) was incubated at every 10°C ranging from 10°C to 100°C for 1 h. Serial dilutions were completed and plated out. The resulting PFU per milliliter from both assays were analyzed on GraphPad Prism 9. This was completed in three biological replicates for each assay.

### Screening for integration of CSP3 into the B. contaminans genome.

In a 10-mL culture, CSP3 was allowed to propagate with B. contaminans overnight at 37°C. The culture was pelleted, the supernatant was discarded, and the remaining bacterial cells were washed 3× with LB broth to remove any potential extracellular CSP3. The cells were then streaked out on LB agar and incubated overnight for single colonies. Colony PCR using OneTaq 2× master mix (NEB) was then performed on these single colonies to detect the presence or absence of CSP3 within the B. contaminans genome. Oligonucleotides (CS128, 5′-GGGCAATATCACGATCACG-3′, and CS129, 5′-GCATTGCTCGTGACGAACG-3′) were made to target CSP3 and were checked for nonspecific binding in B. contaminans. The colony PCRs were run on a gel electrophoresis using 1% (wt/vol) agarose at 80 kV for 40 min and imaged under UV on an Enduro GDS gel documentation system.

### Identification of CSP3 receptor.

The generation and identification of CSP3-resistant mutants were performed as described previously with modifications (83, 84). When plating CSP3 on lawn plates of B. contaminans, no mutants were able to grow through the lysis as previously seen with other phages. Therefore, CSP3 and B. contaminans underwent three rounds of subculturing to allow for time to generate spontaneous resistant mutants. After 3 days, the cultures were pelleted and washed 2× with LB broth. The pellet was streaked out for single colonies and tested against CSP3 to see if they were resistant. Colonies that were no longer sensitive to CSP3 were further purified by restreaking three times and to make sure any mutations were stable. DNA of the mutant strains was extracted using the Wizard genomic DNA purification kit (Promega), and DNA was prepared for Illumina sequencing. The DNA of wild-type B. contaminans was also prepared, sequenced, and assembled with Unicycler v0.4.8 using the default settings (85). SNP analysis was performed with Snippy v4.6.0 using default settings (https://github.com/tseemann/snippy). Mutations identified due to genetic drift or minor error compared to the reference assembly were screened out through SNP comparison between all mutant isolates (i.e., all mutant isolates that equally contained X mutation in Z position were disregarded). Any unique mutations deemed biologically relevant were used for further analysis. All identified mutations (background and unique) are given in Table S1 in the supplemental material. O-antigen gene clusters from B. contaminans 5080 and Bcc strains were done using methods from reference 66.

### Data availability.

The complete genome sequence of the phage CSP3 is available in GenBank under accession number OQ053201.
